# Nitric Oxide Not Apoptosis Mediates Differential Killing of *Mycobacterium bovis* in Bovine Macrophages

**DOI:** 10.1371/journal.pone.0063464

**Published:** 2013-05-15

**Authors:** Hugo Esquivel-Solís, Antonio J. Vallecillo, Alejandro Benítez-Guzmán, L. Garry Adams, Yolanda López-Vidal, José A. Gutiérrez-Pabello

**Affiliations:** 1 Laboratorio de Investigación en Tuberculosis Bovina, Departamento de Microbiología e Inmunología, Facultad de Medicina Veterinaria y Zootecnia, Universidad Nacional Autónoma de México, Mexico City, Mexico; 2 Unidad de Biotecnología Médica y Farmacéutica, Centro de Investigación y Asistencia en Tecnología y Diseño del Estado de Jalisco, A.C. Guadalajara, Jalisco, Mexico; 3 Department of Veterinary Pathobiology, College of Veterinary Medicine and Biomedical Sciences, Texas A&M University, College Station, Texas, United States of America; 4 Programa de Inmunología Molecular Microbiana, Departamento de Microbiología y Parasitología, Facultad de Medicina, Universidad Nacional Autónoma de México, Mexico City, Mexico; University College Dublin, Ireland

## Abstract

To identify the resistance phenotype against *Mycobacterium bovis* in cattle, we used a bactericidal assay that has been considered a marker of this trait. Three of 24 cows (12.5%) were phenotyped as resistant and 21 as susceptible. Resistance of bovine macrophages (MΦ) to BCG challenge was evaluated for its association with *SLC11A1* GT microsatellite polymorphisms within 3′UTR region. Twenty-three cows (95.8%) had a GT_13_ genotype, reported as resistant, consequently the *SLC11A1*polymorphism was not in agreement with our bactericidal assay results. MΦ of cows with resistant or susceptible phenotype were challenged *in vitro* with virulent *M. bovis* field strain or BCG, and nitric oxide production, bacterial killing and apoptosis induction were measured in resting and LPS-primed states. *M. bovis* field strain induced more apoptosis than BCG, although the difference was not significant. Resistant MΦ controlled better the replication of *M. bovis* (P<0.01), produced more nitric oxide (P<0.05) and were slightly more prone to undergo apoptosis than susceptible cells. LPS pretreatment of MΦ enhanced all the functional parameters analyzed. Inhibition of nitric oxide production with *n* ^G^-monomethyl-L-arginine monoacetate enhanced replication of *M. bovis* but did not modify apoptosis rates in both resistant and susceptible MΦ. We conclude that nitric oxide production not apoptosis is a major determinant of macrophage resistance to *M. bovis* infection in cattle and that the influence of *SLC11A1* gene 3′UTR polymorphism is not associated with this event.

## Introduction

Bovine tuberculosis is a disease caused by *Mycobacterium bovis* that was recognized more than 100 years ago and still affects the global cattle industry [1]. Control of bovine tuberculosis is actually driven by the “test and slaughter” strategy, whereby bovine PPD reactor cattle are identified as infected and slaughtered [2]. In addition to the threat for public health, bovine tuberculosis has a significant impact on agriculture causing worldwide annual losses of $3 billion due to depopulation of herds, herd trade restrictions and reduced agricultural productivity, particularly in the developing world and in some developed countries where a residual persistence of infection that cannot be eliminated because of the presence of wildlife reservoirs [3]–[5].

*M. bovis* is an intracellular pathogen that resides mainly in MΦ. Both innate and acquired mechanisms of immunity play important roles in the control of infection. T cell-macrophage interactions have great relevance in the development of protective immunity in bovine tuberculosis and in the generation of immune response parameters utilized in diagnostic tests, such as the specific release and measurement of IFN-γ that has the property to activate MΦ to be more bactericidal [6]. The bactericidal efficacy of MΦ is considered the basis of innate immunity to tuberculosis [7], [8] and a phenotypic marker of cattle resistance to *M. bovis* infection [9].

It is well known that pathogenic mycobacteria induce MΦ to undergo cell death which is modulated by host resistance to mycobacterial infection [10], [11]. Monocytes/MΦ from mice resistant to *Mycobacterium tuberculosis* are more prone to undergo apoptosis after infection than those from susceptible mice that largely die by necrosis [12], [13]. Likewise mouse models, widespread apoptosis of blood monocytes induced by mycobacteria is observed in resistant PPD-positive healthy subjects, as opposed to patients with active TB in which cells are induced to necrosis [14], [15]. Macrophage apoptosis is a host strategy against mycobacteria by depriving the intracellular niches required by bacteria, by limiting bacterial replication and by promoting T cell cross-priming by antigen-presenting cells [16]–[18]. For instance, apoptosis of monocytes infected with *M. bovis* BCG and *Mycobacterium avium-Mycobacterium intracellulare* exposed to ATP^+^ and H_2_O_2_ respectively resulted in loss in viability of intracellular bacilli [19], [20]. Apoptosis is modulated by pathogenic mycobacterial strains and is a correlate of virulence to avoid innate host defense. Virulent strains of *M. tuberculosis* induce more necrosis and less apoptosis than the attenuated/avirulent ones [21]–[23]. Virulent field strains of *M. bovis* induce bovine MΦ to die by apoptosis in a time-dependent manner by a mechanism independent of caspases that is influenced by bacterial load and macrophage soluble factors [24], [25]. TNFα release of bovine activated MΦ by *M. bovis* alone or in combination with LPS or IFN-γ is strongly suggested to play a role in apoptosis induction [6], [26], [27]. Nitric oxide (NO), produced in bovine MΦ [6], [28], plays a major role in the killing of intracellular pathogens by MΦ, and it has also been involved in the regulation of cell death [27], [29]. Previous results from our group have suggested that MΦ with a resistant phenotype, produce more NO and are more prone to undergo apoptosis after *M. bovis* infection than cells with a susceptible phenotype, and that such functional differences are abrogated by the IL-4 effect [30]. In addition, bovine macrophage resistance to intracellular pathogens was initially linked to a polymorphism within the 3′untranslated region of *SLC11A1* (*Nramp1*) gene [31], but eventually was refuted to be associated to *M. bovis* or *Brucella abortus* resistance in cattle with different genetic backgrounds and naturally challenged under field conditions [32], [33].

Altogether, these observations have facilitated us to explore the implication of nitric oxide and apoptosis in the performance of bovine MΦ on resistant (R) and susceptible (S) phenotype. In this study, we demonstrated that R MΦ produced more nitric oxide and were slightly more prone to undergo apoptosis than S cells. The blockade of nitric oxide production enhanced the replication of *M. bovis* in both R an S cells but had no effect on apoptosis induction. We conclude that nitric oxide, not apoptosis, is a major determinant of macrophage resistance to *M. bovis* infection in cattle and that the *SLC11A1* gene 3′UTR polymorphism is not associated in this event.

## Materials and Methods

### Ethics Statement

All animal procedures were performed according to Faculty of Veterinary Medicine of UNAM University (FMVZ-UNAM) board statements on animal research on basis of the Mexican law on animal studies and ethical approval for the study was obtained from the FMVZ-UNAM Institutional Committee for Care and Use of Experimental Animals (CICUAE) (JAGP-2002).

### Animals

Twenty-four Holstein-Friesian females 2 to 7 years old were used in this study as blood cell donors for phenotyping as resistant or susceptible to *in vitro M. bovis* bacille Calmette-Guerin (BCG) infection under macrophage bactericidal assay. Blood samples were collected from the jugular vein of each cow under aseptic conditions using approved safe manual restraint to preserve the animal welfare. All cattle were housed under uniform conditions and nutritional regimens at the Center for Practical Teaching and Research in Animal Production and Health (FMVZ-UNAM, CEPIPSA, Topilejo, Tlalpan, Mexico City.). The cattle were randomly selected from a tuberculosis-free dairy herd (not infected, exposed or vaccinated; status officially declared since 1998) and all animals were negative for the single intradermal comparative cervical tuberculin (SICCT) test for *M. bovis* and *M. avium* antigens.

### Bacteria

We used the attenuated BCG Danish strain and the virulent *M. bovis* 9926 and 129QP field strains isolated from tuberculous lesions of PPD positive cattle. Bacteria were grown at 37°C under shaking conditions in Middlebrook 7H9 broth with 0.05% Tween 80 and supplemented with 10% of OADC enrichment (Becton Dickinson, Cockeysville MD) to mid-log phase to standardize the growth-replication state of cells. BCG strain reached the mid-log phase six days earlier (8th day) than field strains (14th day). Bacteria were suspended in CRPMI, passed twice through a 27-gauge needle and stored at −80°C in 1 mL aliquots. Inoculums were titrated by plating CFU serial dilutions on Middlebrook 7H11 medium (Difco Laboratories, Detroit MI) plus 10% of OADC.

### Bovine Monocyte-derived MΦ

For monocyte isolation, 150 ml of whole blood was collected in acid citrate dextrose (ACD) buffer. MΦ were obtained from peripheral blood monocytes by the method of Campbell *et al*., with slight modifications [34]. Blood mixed with ACD was centrifuged at 1100×*g* for 20 min at room temperature (RT). Buffy coats were suspended with an equal volume of 13 mM sodium citrate in PBS pH 7.4, and carefully layered onto 0.5 volumes of Percoll suspension (Pharmacia, Uppsala, Sweden) at specific density of 1.077 adjusted with 130 mM trisodium citric acid, 5% BSA and PBS. Layers were centrifuged at 1000×*g* for 30 min at RT. Leukocytes were recovered and washed three times with 5% autologous plasma in PBS citrate for 10 min at 350×*g* and resuspended at 5×10^6^ cells/ml in CRPMI (RPMI 1640 plus 2 mM L-glutamine, 0.1 mM non-essential aminoacids, 1 mM sodium piruvate and 20 mM sodium bicarbonate) (Gibco BRL, Life Technologies, Grand Island, NY) containing 4% autologous serum, and 5 ml were placed into 50 ml Teflon flasks (Nalgene, Nalge Nunc, Naperville IL) and cultured for 4 h at 37°C and 5% CO_2_. Non-adherent cells were removed by three washes with pre-warmed PBS, and adhered monocytes were cultured at the above conditions in CRPMI with 12.5% autologous serum for 12 days. MΦ were harvested by repeated gently pipetting after chilling flasks on ice for 45 min and suspended at densities indicated on each experiment. Macrophage priming was achieved by culturing cells with 100 ng/mL of Lipopolysaccharide (LPS) from *E. coli* O26:B6 (Sigma Chemical Company, St. Louis, MO) in CRPMI plus 12% of autologous serum for 22 h previous to infection with *M. bovis*. Blocking nitric oxide production was achieved by culturing cells with 1 mM of *n* ^G^-monomethyl-L-arginine monoacetate (MMLA, Sigma Chemical Company, St. Louis, MO).

### Macrophage Infection and Macrophage Bactericidal Assay

MΦ were infected with BCG or *M. bovis* virulent strains for 4 h at a multiplicity of infection (MOI) of 10 mycobacteria (CFU) per macrophage and centrifuged at 200×*g* for 10 min. Macrophage bactericidal assay was performed with 1×10^4^ MΦ per well in Nunc MiniTrays (Nalge Nunc International, Rochester, NY) [9]. After 4 h, non-ingested and non cell-attached bacteria were removed by four washes with RPMI. Initial count of mycobacterial uptake was quantified by plating serial dilutions of cell suspension after lysis with 0.5% Tween 20. Intramacrophage mycobacterial growth was assessed at 24 h postinfection. The number of ingested bacteria was calculated by dividing total CFU by total MΦ. Mycobacterial intracellular survival was represented by mycobacterial growth index and was calculated by dividing the number of CFU at end of assay (24 h) by the number of CFU at start of assay (0 h) expressed as percentage. A value of 65% or less of bacterial survival for *M. bovis* BCG has been correlated with actual numbers of cattle designated as resistant (R), while higher values correlate with designation of susceptible (S) and therefore is considered a phenotypic marker of the resistant trait [9]. MΦ from the three resistant and three susceptible cattle were used for further functional comparative analysis.

### Genotyping of *SLC11A1*(*Nramp1*) 3′UTR Polymorphism

Blood samples collected into tubes containing EDTA were used for genomic DNA extraction and genotyping. Genotyping of all 24 cows was performed by Single Stranded Conformation Analysis (SSCA). One hundred ng of genomic DNA, was amplified using primers specific for the 3′ untranslated region of the bovine *SLC11A1* (*Nramp1*) gene (nucleotide positions 1814–1989). The forward primer (5′-AAGGCAGCAAGACAGACAGG-3′) and reverse primer (5′-ATGGAACTCACGTTGGCTG-3′) were used as previously described [33]. PCR was performed as previously described [33]. The PCR products were denatured at 94°C for 5 min in formamide solution (95% formamide, 10 mM EDTA pH 8.0, 0.5% Xilene cyanol, 0.5% bromofenol blue) (Sigma Chemical CO, St Luis,MO), run in a 6% acrylamide gel at 55 V for 150 min, and silver stained (Silver Stain Plus kit, cat. 161-0449, BioRad, Hercules, CA) on the gel. DNA samples of genotypically resistant and susceptible cattle to standardized virulent *Brucella abortus in vivo* challenge were used as controls for the assay [35]. DNAs of two susceptible and two resistant cows were subcloned and sequenced.

### Measurement of Nitric Oxide and Apoptosis

Nitrite (NO_2_), as an indicator of NO production, was determined in 100 µl cell culture supernatants of 5×10^4^ MΦ with 100 µl of Griess reagent (Sigma Chemical Company, St. Louis, MO). NO_2_ content was calculated from a NaNO_2_ standard curve. After 15 min the absorbance at 540 nm (A_540_) was measured. For quantitative determination of apoptosis induction, chromatin condensation was measured at 8, 16 and 24 h and DNA fragmentation at 16 and 24 h postinfection. Triplicates of 2×10^5^ MΦ adhered to 12 mm diameter round-glass coverslips cultured in 24-well tissue culture plates and stained with Propidium iodide (5 µg/mL and 150 µg/mL Ribonuclease A) (Sigma Company, St. Louis, MO) were used to enumerate chromatin condensed nuclei from 200 cells per coverslip using an epifluorescent microscope (Leica, Microstar IV). Results are the mean ±1 SD in percentage from triplicates of three independent experiments. An aliquot of 10^6^ cells cultured in 50 mL Teflon flasks was used to count DNA fragmented nuclei by the TUNEL assay with the APO-BRDU kit (BD Biosciences, San Jose, CA) following manufacturer instructions. All cells were stained in the presence or absence of TdT enzyme to control the specificity of the assay. The number of fluorescein thiocyanate (FITC)-positive cells was acquired by counting 1×10^4^ cells using a Flow Cytometer (Coulter, EPICS Altra). Data were analyzed with Expo Altra v.2 software. Results are presented as histograms of total cell counts versus FITC signal in log_10_ scale. Values are the percentage of Bromo-deoxi-Uridine (BrdU)-FITC-positive cells. Camptothecin-treated (10 µg/mL for 48 h) MΦ were used as control of positive apoptotic features, whereas non-infected MΦ were employed as negative controls.

### Statistical Analyses

The association between phenotype and allele polymorphism of the 3′UTR *SLC11A1* (*Nramp1*) gene was verified by Fisher’s exact test. Differences among susceptible and resistant phenotypes and between *M. bovis* strains were tested for statistical significance by one-way ANOVA. All tests were performed using GraphPad Prism version 5.00 for Windows (GraphPad Software, San Diego California USA, www.graphpad.com). A *p* value equal or less than 0.05 was considered to be statistically significant.

## Results

### Herd Screening for Resistant Cattle through Macrophage Bactericidal Response

**Figure 1:**
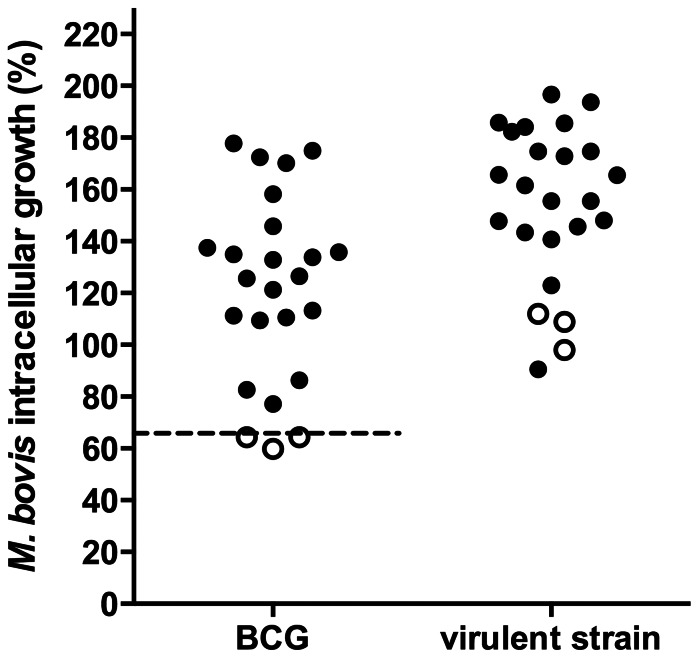
Bovine macrophage BCG growth control as an indicatior of a natural disease resistance phenotype. Adherent-macrophages were infected with BCG Danish strain or *M. bovis* field strain 9926 (MOI of 10) for 4 h, washed and cultured again for 24 h. Bacterial intracellular growth was calculated by dividing the number of intracellular CFU at 24 h post-infection by the number of intracellular CFU at the start of the assay. The figure shows the percentage of *M. bovis* growth in macrophages of 24 cows. For BCG values below or equal to the cut-off point of 65% growth (dotted line) are indicative of resistant cattle (white symbols) while values higher than 65% are a sign of susceptibility (black symbols). Plots are mean of triplicates of two independent experiments of each animal.

According to Qureshi T, *et al*. a cut-off point of 65% of BCG survival was established to designate an animal as resistant (R) or susceptible (S). In our study, MΦ of 3/24 cattle (12.5%) restricted BCG intracellular replication on levels equal or lesser than 65% thus they were classified as R, otherwise cattle were classified as S (21/24; 87.5%). Virulent *M. bovis* grew to a larger extent in all bovine MΦ but R MΦ were more efficient in controlling intracellular growth than S cells (*P*<0.05) (Figure 1). These differences were not related to phagocytosis since all MΦ ingested a similar number of mycobacterial UFC regardless of their phenotype (data not shown).

### *SLC11A1* (*Nramp1*) 3′Untranslated Region Polymorphisms and their Association with Resistance to *M. bovis* in Cattle

**Figure 2:**
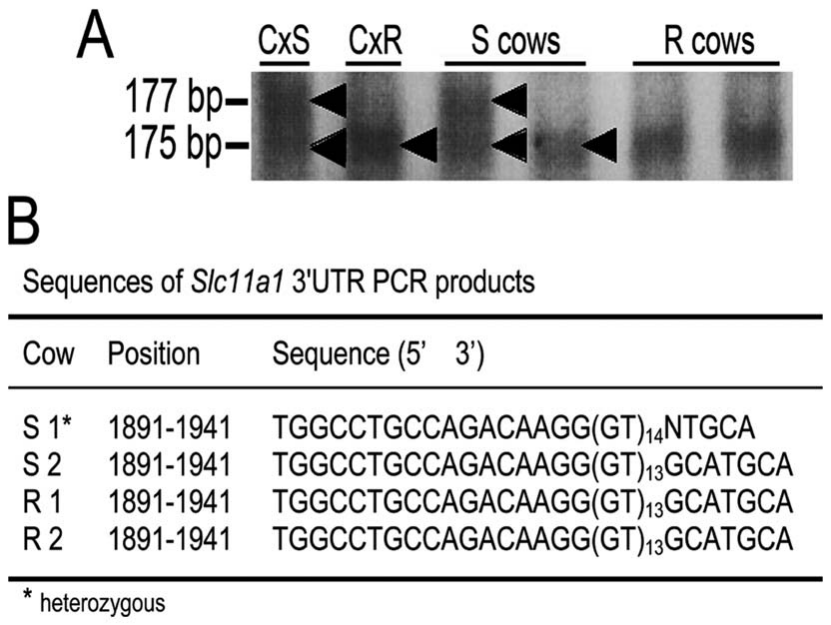
3′ UTR *SLC11A1* gene polymorphism analysis in resistant and susceptible macrophages. Representative results from resistant (R) and susceptible (S) cattle genomic DNA single-stranded conformational analysis (SSCA) indicating the homozygous GT13 (175 bp) and heterozygous GT13/GT14 (177 bp) alleles. DNA from cattle genetically characterized as susceptible (SLC11A1s) or resistant (SLC11A1r) to standardized *Brucella abortus* in vivo challenge were used as controls (Cx) (A). DNA sequencing of PCR amplicons of *SLC11A1* 3′UTR confirmed the SSCA results evidencing the only one heterozygous genotype among all phenotyped susceptible and resistant cattle to BCG (B).

Resistance of bovine MΦ to BCG challenge was evaluated for the association with *SLC11A1* GT microsatellite polymorphisms within 3′UTR region. SSCA results showed only one susceptible genotype among all 24 cows analyzed, corresponding to the heterozygous GT_13_/GT_14_ microsatellite repeats in 3′untranslated region confirmed by DNA sequencing (Figure 2). Given the proportion of susceptible (21/24) and resistant (3/24) phenotypes into the herd and the very small frequency of susceptible genotype (1/24) among the same sample population, it was obvious that there is no association between the *SLC11A1*-resistant allele and the *SLC11A1*-susceptible allelle with the resistant or susceptible phenotypes, respectively (Table 1).

**Table 1:** Frequencies of *SLC11A1^s^* and *SLC11A1^r^* genotypes among cattle characterized as susceptible or resistant by BCG killing assay.

	genotype	
phenotype^a^	*SLC11A1^s^*	*SLC11A1^r^*
susceptible (n = 21)	1	20
resistant (n = 3)	0	3

a Fisher’s exact test reveals no association between macrophage phenotype and the 3′UTR GT-polymorphism in *SLC11A1* gene.

### Nitric Oxide Production by *M. bovis*-infected R and S Bovine MΦ

**Figure 3:**
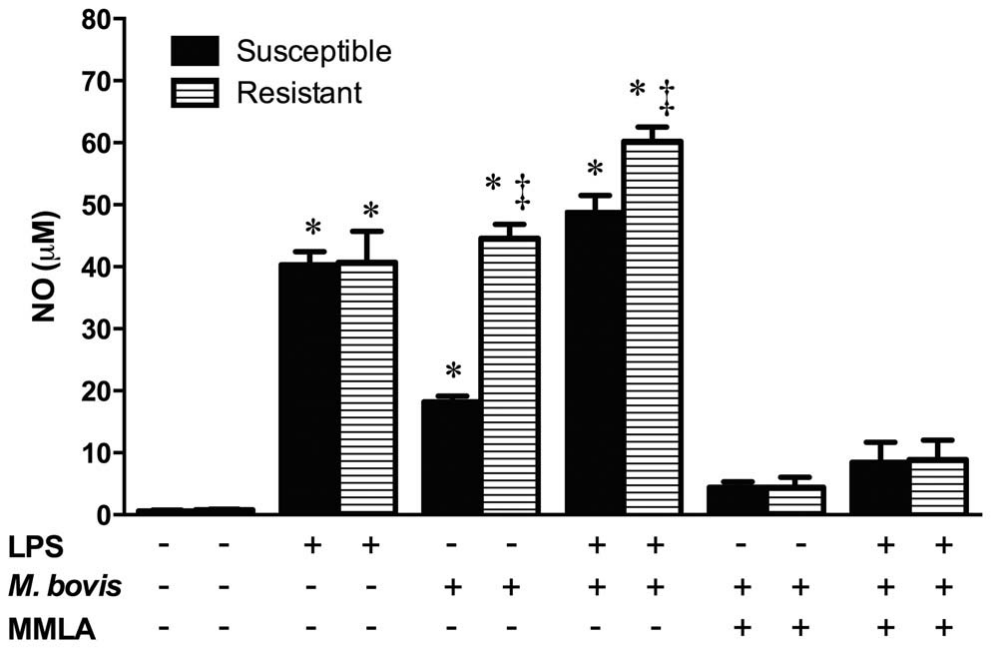
*Mycobacterium bovis* nitric oxide differential induction in susceptible and resistant bovine macrophages. Macrophages stimulated or not with purified *E. coli* 026:B6 LPS (100 ng/mL) for 22 h, were infected with *M. bovis* field strain 129QP (MOI of 10) for 4 h. Cells were washed and cultured again for 24 h in presence or absence of the nitric oxide inhibitor *n* ^G^-monomethyl-L-arginine monoacetate (MMLA) and nitric oxide was measured in cell culture supernatants by Griess assay. Results are mean ± standard deviation of quadruplicates of two independent experiments with macrophages of three susceptible and three resistant cows. Statistical differences (*P*<0.05) of each bar with its respective control (no treatment) (*) and among phenotypes on each condition (‡) are indicated.

Infection of bovine MΦ with BCG or virulent *M. bovis* revealed a functional difference in controlling growth of both strains among cattle. Levels of NO released in response to *M. bovis* were significantly increased compared to levels released by uninfected cells (∼1 µM), but were much higher in R (∼45 µM) MΦ than in S cells (∼18 µM) (p<0.05). LPS pretreatment, used as an unbiased stimulus of iNOS, induced large NO release (∼40 µM) in R and S MΦ without revealing any differences between them, however LPS pretreatment followed by *M. bovis* infection revealed a higher NO production by the R (∼60 µM) MΦ compared to S cells (∼49 µM) without significant differences (p>0.05). Neutralizing NO production with MMLA in *M. bovis* and/or LPS treatments confirmed that NO production was dependent and specific for the stimulus (Figure 3).

### Nitric Oxide and Macrophage Resistance to *M. bovis in* Cattle

**Figure 4:**
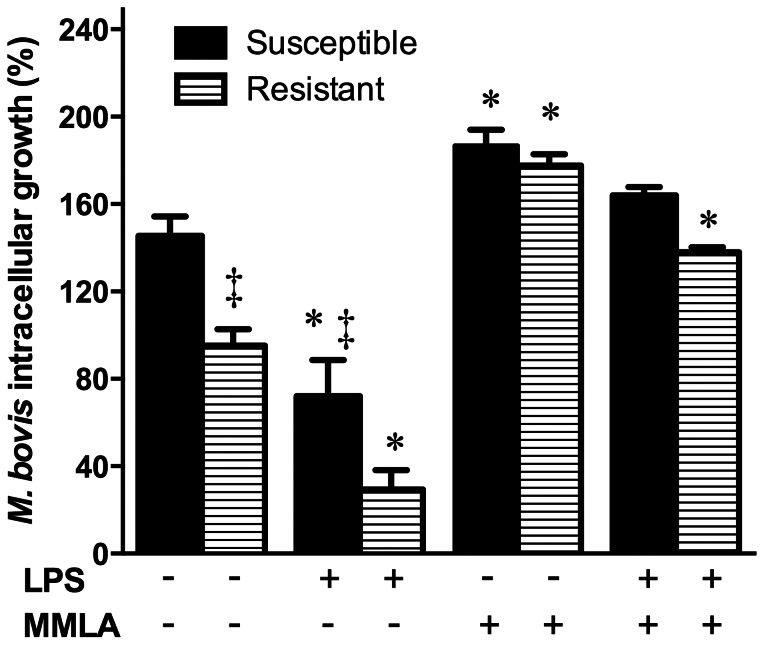
Control of *Mycobacterium bovis* growth control in bovine macrophages is related to nitric oxide production. Adherent-macrophages stimulated or not with purified *E. coli* 026:B6 LPS (100 ng/mL) for 22 h, were infected with *M. bovis* field strains 9926 or 129QP (MOI of 10) for 4 h, washed and cultured again for 24 h in presence or absence of *n* ^G^-monomethyl-L-arginine monoacetate (MMLA). Bacterial intracellular growth was calculated by dividing the number of intracellular CFU at 24 h postinfection by the number of intracellular CFU at the start of the assay and expressed as percentage. Values are mean ± standard deviation of triplicates of two independent experiments with macrophages of three susceptible and three resistant cows and challenged with each field strain. Statistical differences (*P*<0.05) of each bar with its respective control (no treatment) (*) and among phenotypes on each condition (‡) are indicated.

NO production by R and S bovine MΦ was *M. bovis* and/or LPS dependent, but the differences were significant only in response to *M. bovis*. NO amount was related to the macrophage microbicidal phenotype. We sought to determine if *M. bovis* replication was in a direct relationship with NO production by MΦ. Enhancing NO production with LPS stimulus (as shown in figure 3) significantly decreased *M. bovis* replication in MΦ (Fig. 4), with higher reduction in R MΦ (3 fold reduction, from 95% to 29% bacterial survival) than in S cells (2 fold reduction: from 145% to 72% bacterial survival) (p<0.05). Blocking NO by the addition of MMLA in *M. bovis* infected MΦ substantially reduced their inherent ability to restrict *M. bovis* replication (Fig. 4), resulting in a ∼2 fold increase in bacterial survival in R MΦ (from 95% to 177%; p<0.05). However, R MΦ pre-stimulated with LPS and NO blocked with MMLA had a reduced increase in *M. bovis* replication (∼1.4 fold, from 95% to 138%; p>0.05) while resting or LPS-stimulated S MΦ had only 1.29 and 1.13 fold increases respectively (from 145% to 187% and 164% bacterial survival; p>0.05) (Fig. 4).

### Macrophage Apoptosis and *M. bovis* Resistance

**Figure 5:**
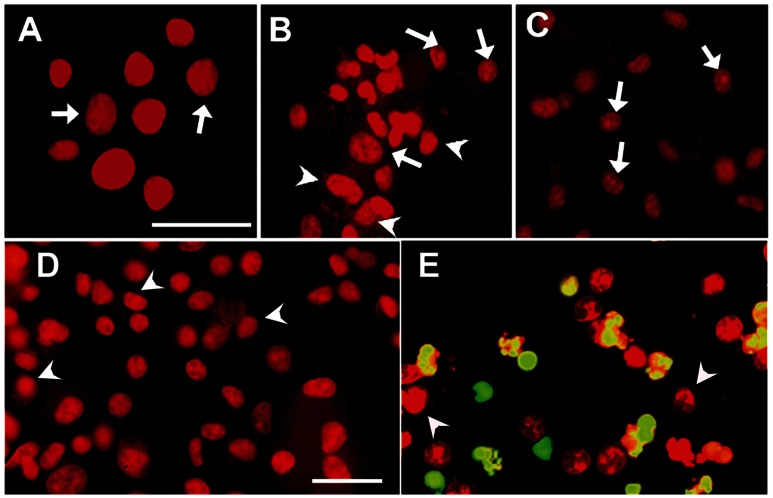
Apoptosis induction in *Mycobacterium bovis*-infected bovine macrophages. Uninfected macrophages (A), macrophages treated with camptothecin (10 µg/mL for 48 h) (C) and macrophages infected with *Mycobacterium bovis* field strain 9926 (B, D and E), were washed at 4 h and cultured again for 24 h. Then cells were processed by TUNEL in presence (E) or absence (D) of TdT enzyme. Chromatin condensation was present in no-infected (arrows) and infected cells (arrowheads). TUNEL-positive cells show a green-yellow mark in panel E (BrdUTP-FITC). Magnification for A to C is 63X and for D and E is 40X with a scale bar of 50 µm each.

**Figure 6:**
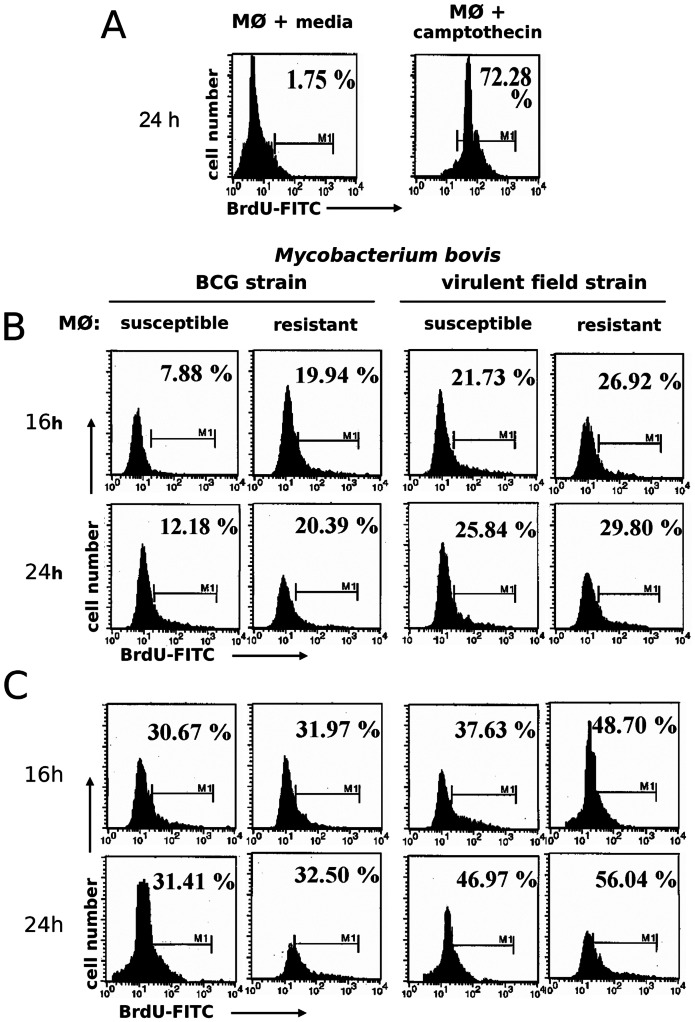
*Mycobacterium bovis* induction of bovine macrophage DNA fragmentation is time dependent. Adherent-macrophages (MØ) were cultured with media alone or with camptothecin (10 µg/mL for 48 h) as negative and positive controls, respectively (A). Also macrophages were cultured in absence (B) or presence of purified *E. coli* 026:B6 LPS (100 ng/mL) for 22 h (C), and infected with BCG or *M. bovis* field strain 9926 (MOI of 10 for 4 h), washed and cultured again for 16 and 24 h and stained with TUNEL (BrdUTP-FITC). Histograms are frequency distributions of 1×10^5^ macrophages along FITC signal (log_10_ scale). Values are percentages of true BrdU-FITC TUNEL-positive cells (M1 gate) of one experiment, representative of two independent experiments with macrophages of three susceptible and three resistant cows. One-way ANOVA showed significant variation in infected versus uninfected controls (*P*<0.05) but not between phenotype (*P* = 0.4544) regardless of *M. bovis* strain.

Many more cells with chromatin condensation were observed in *M. bovis*-infected MΦ than uninfected cells, an indication of apoptosis induced by *M. bovis* that was confirmed by specific labeling of DNA fragmentation (nick ends) by TUNEL (Figure 5). Given that we observed differences in MΦ controlling *M. bovis* replication, we sought to determine any correlation of apoptosis with mycobacterial control by comparing proportions of R and S *M. bovis*-infected MΦ with apoptosis. Numbers of MΦ with chromatin condensation were significantly higher in R MΦ than those in S cells upon 24 h infection with *M. bovis* (R = 78%, 88% 94% vs. S = 47%, 58%, 68% at 8, 16 and 24 h, respectively; p<0.05) or BCG (R = 32%, 52%, 61% vs. S = 14%, 22%, 34% at 8, 16 and 24 h, respectively; p<0.05) (Table 2). The pre-treatment with LPS was associated with enhanced numbers of cells with chromatin condensation in both R and S *M. bovis*-infected MΦ and eliminated the differences among them (p>0.05) (Table 2). Given that DNA fragmentation is a hallmark of apoptosis and that it follows chromatin condensation, we measured DNA fragmentation with TUNEL in R and S *M. bovis*-infected MΦ by flow cytometry at 16 and 24 hours post-infection. The number of MΦ that underwent DNA fragmentation was higher in R MΦ than S MΦ at16 h and 24 h post-infection with *M. bovis* and BCG, however these differences were not statistically significant, nor were the differences between macrophage phenotypes (R vs. S) between virulence of bacteria (*M. bovis* vs. BCG), or between resting and LPS-stimulated MΦ (p>0.05 in all comparisons) (Figure 6).

**Table 2:** Kinetics of apoptosis induction in resistant and susceptible *Mycobacterium bovis*–infected bovine macrophages, expressed as percentage (mean ± SD) of cells showing chromatin condensation after infection.

Phenotype	Pre-treatment	*M. bovis*	8 h^a^	16 h	24 h
Susceptible	–	–	4.3±2.3	4.2±1.5	5.5±1.5
		BCG	14.0±1.7	22.3±1.7	34.2±3.8
		field strain	47.7±3.9	58.5±2.3	68.0±1.9
	LPS	–	11.3±1.5	16.3±3.1	19.8±3.6
		BCG	35.0±3.0	53.8±3.6	90.1±4.2
		field strain	52.0±4.0	68.0±3.2	95.0±2.9
Resistant	–	–	4.6±1.30	2.2±0.7	4.2±0.3
		BCG	32.7±4.7*	52.7±1.6*	61.8±1.7*
		field strain	78.2±5.3*	88.5±3.5*	94.7±2.6*
	LPS	–	12.7±3.2	12.2±0.7	17.7±2.9
		BCG	47.5±1.9	86.7±2.7*	85.5±1.3
		field strain	88.1±0.4*	94.5±2.5*	99.0±0.6

a Macrophages were pre-cultured with or without LPS for 18 h then infected with BCG or *M. bovis* field strain for 4 h, washed and cultured again for indicated times and then stained with propidium iodide. Two hundred macrophages were analyzed per slide in triplicate cultures and those with chromatin condensation were counted. Results are representative of two independent experiments with macrophages of three susceptible (S) and three resistant (R) cattle.

* Significant differences (*P*<0.05) are among phenotypes.

### Nitric Oxide Effect in Apoptosis Induction of *M. bovis*-infected Bovine MΦ

**Figure 7:**
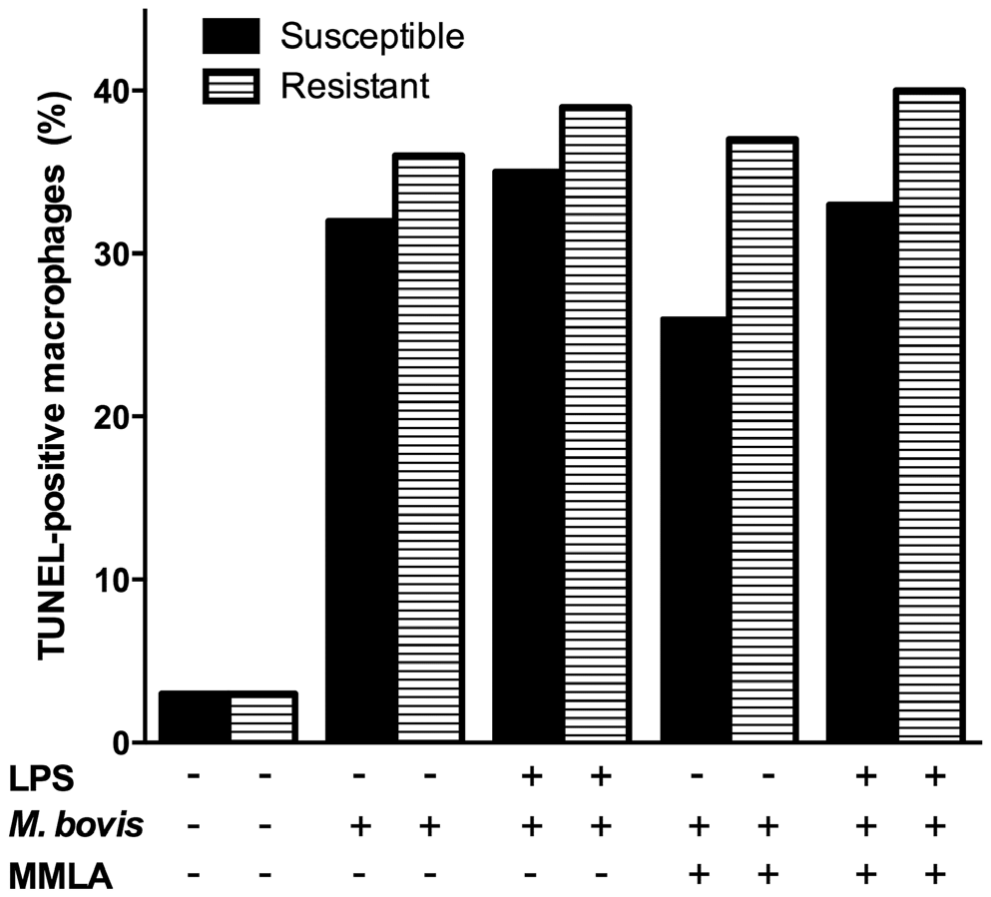
*Mycobacterium bovis* bovine macrophage apoptosis induction is nitric oxide independent. Adherent-macrophages stimulated or not with purified *E. coli* 026:B6 LPS (100 ng/mL) for 22 h, were infected with *M. bovis* field strain 9926 (MOI of 10) for 4 h, washed and cultured again for 24 h in presence or absence of *n* ^G^-monomethyl-L-arginine monoacetate (MMLA) and stained with TUNEL (BrdUTP-FITC). Values are percentages of true BrdU-FITC TUNEL-positive cells of one experiment, representative of two independent experiments with macrophages of three susceptible and three resistant cows. Kruskal-Wallis test showed significant variation versus uninfected controls (*P*<0.05) but not between treatments (*P* = 0.4373) regardless of macrophage phenotype.

Induction of apoptosis in *M. bovis*-infected bovine MΦ was associated with the release of high levels of nitrite in the culture medium at 24 h post-infection. Given that bovine MΦ differentially controlled *M. bovis* replication in a NO dependent fashion and that the proportion of macrophage undergoing apoptosis agreed with NO levels, we sought to determine the association of NO with apoptosis induction of *M. bovis*-infected MΦ. Blocking NO by the inclusion of MMLA did not modify the response of R or S MΦ to die upon *M. bovis* infection (Fig. 7).

## Discussion

Infection with *M. bovis* is a major health problem causing zoonoses and economical losses in cattle industry in many countries [2], [36]. *M. bovis* infects and replicates within the hostile intracellular environment of MΦ as a mechanism of persistence by avoiding the immune system, or as a mechanism of dissemination to other cells and tissues by inducing macrophage to die by necrosis [37], therefore the response elicited by MΦ after the encounter of *M. bovis* is crucial in determining the outcome with infection whereby they are potentially involved in innate resistance of cattle to tuberculosis.

Limited information is available on the inherent ability of MΦ to control *M. bovis* replication through bactericidal products (NO) and other mechanisms implicated in resistance to infection, and relatively little is known about the differences of these factors between MΦ having a differential control over *M. bovis.* We used the innate macrophage ability to restrict BCG replication as a correlate with natural resistance to intracellular pathogens measured by a microbicidal assay [9] in order to select resistant and susceptible cattle as cell donors for the experiments. Using this assay in a sample of a dairy herd (*n* = 24 cows), we identified 3/24 (12.5%) with a phenotype of resistance. The proportion of cattle detected as resistant in our experimental *in vitro* conditions using microbicidal assay, agree with the frequency of natural disease resistant cattle detected at slaughter, supporting the usefulness of the assay [33]. In that study, cattle were tuberculin-skin test negative animals that lived in herds with naturally-occurring bovine tuberculosis which were constantly exposed to *M. bovis* but no tuberculosis-like lesions were observed at necropsy and the *M. bovis-*culture was negative [38].

A microsatellite (GT)13 repeat at the 3′UTR of bovine *SLC11A1* (*Nramp1*) gene was initially related with resistance of cattle to *M. bovis* and other intracellular pathogens [35], [39]. By further investigation of the *M. bovis* bactericide response in MΦ, we were able to identify improved phenotyping of previously selected R and S cattle. SSCA indicated that homozygous alleles with microsatellite lengths of 13 GT dinucleotide repeats (resistant genotype) were found in all three cows resistant to *in vitro* challenge with BCG matching perfectly, while heterozygous alleles with microsatellite lengths of 13/14 GT dinucleotide repeats (susceptible genotype) were found in only one of the 21 cattle susceptible to *in vitro* challenge by *M. bovis* BCG, while the other 20 susceptible cattle had a resistant genotype, therefore we were unable to assign the correct phenotype to the genotype in S cattle under our conditions. One possible reason for this is that we selected a phenotype based on the performance of the microbicidal assay not the genotype of our dairy herd. Previous standardized *in vivo* wild type *B. abortus* challenge studies in unvaccinated, unexposed 180 day pregnant first calf heifers revealed that microsatellite lengths of 13 GT dinucleotide repeats were found in cattle resistant to challenge by *B. abortus*, while microsatellite lengths of 14, 15, and 16 GT dinucleotide repeats were found in cattle susceptible to *B. abortus* challenge. However a minority cattle with ≤ 13 GT were susceptible, and a majority ≥ 14 GT cattle were resistant, thus the association of natural resistance was not perfect for the GT polymorphisms of *SLC11A1* in these studies [35]. The same conclusion was drawn in other studies on polymorphisms within this region of the gene that from cattle of different genetic backgrounds were not associated with disease resistance to *B. abortus* or *M. bovis* in cattle under field conditions [32], [33], [40]. The explanation maybe the discrepancies of standardized (a single specific CFU dose) vs. natural field (discontinuous dose and multiple challenges) challenge differences in assigning phenotypes vs. genotypes, rather than failing of the gene in supporting resistance. Analysis of immune correlates related to natural resistance to *Brucella* revealed a differential response in macrophage activation between resistant and susceptible cattle, along with the associated segregation of specific alleles of bovine *SLC11A1* as observed in R cattle in the present study.

The frequency of natural resistance to brucellosis was shown to be 18% in cross-bred cattle [41]. Although we did not screen a large number of cattle, our results identified 12.5% (3/24) of cattle with the resistant phenotype suggesting that the percentage of natural disease resistance against *Mycobacterium bovis* in cattle is not high as well, thus limiting the possibility of identifying R animals. In this study, we included the three R individuals available and matched with an equal size sample of S animals for functional comparisons on the basis that they all were of the same breed, similar age and belonged to the same herd with the same housing conditions and that no substantial differences were detected. In addition, three independent replicas were performed for each experiment, each one including three internal repetitions to reduce the impact of individual and experimental variations. The mechanisms involved in differential control of *M. bovis* between R and S MΦ were investigated by comparing responses under these circumstances. We primarily identified the role of NO in the *M. bovis* killing response of resistant MΦ. It was previously shown that NO generated by *M. bovis* infection in bovine MΦ do not reach levels that affect mycobacterial replication [6], [42]. Previous works also indicated the requirement of priming with an individual or combinatory stimulus of IFN-γ and LPS to block the replication of mycobacteria, but the effect apparently is not determined by the release of NO, as neutralizing NO, via the inclusion of the inhibitor MMLA, had no impact on the intramacrophage replication of mycobacteria [6], [43]. We observed that NO release was substantially induced in LPS-primed MΦ alone or after *M* bovis infection with significantly higher levels in resistant compared to susceptible MΦ. This suggested that NO generation is a key process in anti-mycobacterial activity in resistant MΦ. Indeed priming with LPS enhanced macrophage resistance to *M. bovis* infection which was abrogated by blocking NO synthesis with MMLA incorporation, and rendered resistant cells as permissive as untreated control cells and also transformed susceptible cells to an even more permissive state, suggesting that additional differential factors are involved in mycobacterial replication blocking than only NO. In addition, LPS had no effect on bacterial uptake in cells. These findings are partially in disagreement with data generated in previous reports about the role of NO in blocking *M. bovis* replication [6], [43]. The situation is more complex in functional studies of cells involving intrinsic phenotypic differences. Although our data suggest that phenotypic resistance against *M. bovis* is strongly associated to NO release, as a mechanism of generation of maximal bactericidal activity, other possible soluble factors as TNF-α or other mechanisms including iron deprivation [20], [44], [45] may be involved, however their role in bacterial killing require further evaluation in the bovine model.

In our system, MΦ with enhanced mycobactericidal activity also had enhanced chromatin condensation, whereas those that allowed *M. bovis* replication had fewer apoptotic counts. This suggested a role for macrophage apoptosis in macrophage resistance to *M. bovis* and a link with NO release. Pathways of apoptosis induction in MΦ via the generation of NO have been described [26]. The exact bactericidal mechanisms activated for apoptosis in the infected macrophage remain undefined, but may be related to calcium and ATP dependent effects [17], [20].

Previous works have suggested that induction of bovine macrophage apoptosis is closely linked to the emergence of macrophage resistance to *M. bovis* replication, which is dependent on TNF-α release [6], [30]. Virulent *M. bovis* has been shown to induce release of higher levels of pro-inflammatory mediators by bovine MΦ, compared to levels released upon BCG infection. Among these mediators are TNF-α, IL-12 and NO, whose release is significantly enhanced by IFN-γ prior to infection and by bovine Natural Killer cells direct contact [6], [43]. We previously reported that bovine macrophage apoptosis is induced by live *M. bovis* and by its cellular components [24], [25].

It has been shown that pathogenic mycobacteria induce less apoptosis in monocytes/MΦ than avirulent mycobacteria, as a mechanism of evasion of host immune response. Virulent *M. tuberculosis* induces less apoptosis and more necrosis of human monocyte/MΦ than attenuated *M. tuberculosis* H7Ra or BCG [10], [11], [23]. Rojas *et al*. (1997) showed that avirulent *M. tuberculosis* H37Ra induced lesser macrophage death and NO production than virulent *M. tuberculosis* H37Rv in mouse MΦ [12]. These data provide more evidence that macrophage apoptosis is a mechanism of innate resistance to mycobacterial infection. It has been recently found that after apoptosis of *M. tuberculosis*-infected MΦ, the apoptotic cell debris harboring the bacterium are rapidly taken up by uninfected MΦ and delivered to the lysosomal compartment and *M. tuberculosis* is killed, indicating that apoptosis itself is not intrinsically bactericidal but facilitates presentation of bacteria sequestered within an apoptotic body to be presented to other MΦ, thus indirectly functioning as a microbicidal mechanism [46].

The role of macrophage apoptosis in immune resistance against mycobacteria has been studied in great detail in mouse models. Genetically resistant mouse MΦ responses to a standardized challenge with intracellular pathogens (B10R, *Nramp* ^+/+^, *sst1^R^*), exhibit apoptosis after *M. tuberculosis* infection at superior levels than susceptible MΦ (B10S, *Nramp* ^−/−^, *sst1^S^*) and correlate with generation of NO and TNF-α and demonstrate better restriction of *M. tuberculosis* [12], [13], [47]. In addition, people who are infected with *M. tuberculosis* but do not develop active disease exhibit higher apoptosis than necrosis of monocytes/MΦ induced by *M. tuberculosis* or its components compared to people who progress to a clinical outcome and experience more necrosis than apoptosis [14], [15].

In our natural field model, for all conditions studied resistant MΦ had higher apoptosis values than susceptible ones, however only a few were significantly superior as were in controlling intracellular growth of *M. bovis*. Although our data suggested a relationship between NO release and apoptosis induction, blocking NO by inclusion of MMLA did not abrogate apoptosis induction by *M. bovis* neither in resistant nor in susceptible MΦ. Even though macrophage apoptosis was induced at a different level in susceptible and resistant MΦ, the role in macrophage resistance to *M. bovis* was inconclusive and requires further definition. In conclusion, our results strongly suggest a key role for NO in bovine macrophage resistance to *M. bovis*.
